# From inflammaging to steroid resistance: reframing the pathogenesis of neutrophilic asthma

**DOI:** 10.3389/falgy.2026.1859908

**Published:** 2026-07-15

**Authors:** Lei Li, Moping Sun, Yibo He, Xinning Liu

**Affiliations:** 1 Department of Acupuncture Massage & Rehabilitation, Qingdao Traditional Chinese Medicine Hospital, Qingdao Hiser Hospital Affiliated of Qingdao University, Qingdao, Shandong, China; 2 Emergency Medicine Center, Qingdao Traditional Chinese Medicine Hospital, Qingdao Hiser Hospital Affiliated of Qingdao University, Qingdao, Shandong, China; 3 Department of Clinical Pharmacy, Qingdao Traditional Chinese Medicine Hospital, Qingdao Hiser Hospital Affiliated of Qingdao University, Qingdao, Shandong, China

**Keywords:** aging, inflammaging, neutrophilic asthma, steroid, resistance, neutrophil chemotaxis

## Abstract

Asthma is a chronic respiratory disease characterized by airway obstruction, persistent inflammation, and tissue remodeling. Among its subtypes, neutrophilic asthma (NA) is particularly challenging due to its high severity and glucocorticoid resistance. NA is primarily characterized by T2-low (non-T-helper cell type 2-driven) airway inflammation, driven by a network of mediators—including IL-6, IL-8, IL-17, IL-1β, TNF-α, and IFN-*γ*—which act in concert to orchestrate neutrophil recruitment and perpetuate chronic neutrophilic inflammation. Currently, NA lacks defined therapeutic targets, largely due to an incomplete understanding of its pathogenesis. In our previous study, transcriptomic analysis revealed that the pathogenesis of NA is closely associated with aging. Specifically, the accumulation of aging-related cells releases the senescence-associated secretory phenotype (SASP), which appears to play a pivotal role in establishing and amplifying neutrophilic inflammation. Disrupting the pathological loop orchestrated by cellular aging (“aging–inflammation amplification–steroid resistance”) thus emerges as a compelling therapeutic rationale. In this article, we systematically explore the complex signaling networks mediating the interplay between aging and NA, aiming to provide new theoretical insights and research directions for the treatment of this refractory asthma subtype.

## Graphical Abstract

**Figure:**
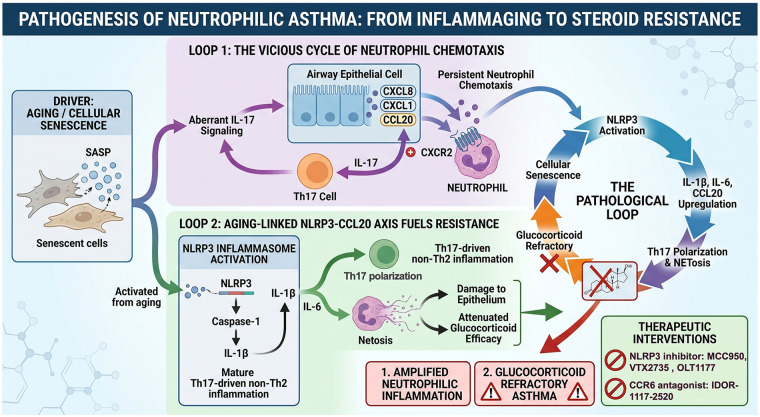

## Aging and the pathogenesis of NA

Cellular senescence is characterized by irreversible cell cycle arrest, typically accompanied by accumulated DNA damage and the formation of a pro-inflammatory microenvironment. A central effector of senescence is the SASP. Senescent cell accumulation in the lungs induces oxidative stress via SASP, promotes chronic inflammation, disrupts tissue regeneration, and delays airway recovery (1, 2). This age-associated process correlates with a higher incidence of asthma, which increases significantly in individuals over 60 (3, 4). Moreover, intrinsic epigenetic age acceleration exhibits a bidirectional causal relationship with allergic asthma (5), suggesting that molecular mechanisms of senescence play a critical role in asthma pathogenesis.

Extensive studies have highlighted the role of neutrophils in elderly asthma, revealing that with advancing age, airway neutrophil counts and proportions significantly increase, accompanied by a decline in innate immune function and impaired anti-inflammatory responses (6–8). In individuals over 70, these alterations are more pronounced, manifesting as increased bronchial neutrophilic infiltration, reduced lung function, and poorer asthma control—often requiring higher doses of inhaled corticosteroids (ICS), greater dependence on oral corticosteroids (OCS), and more frequent asthma-related hospitalizations (9). Comparative murine studies using HDM-induced asthma models have revealed that, relative to young mice, aged mice exhibit weakened type 2 and heightened, sustained type 3 (Th17/neutrophilic) immune responses—indicating age-related immune remodeling toward neutrophilic inflammation (10).

Despite these observations, few studies have directly associated NA with molecular mechanisms of aging or cellular senescence. A 2025 review on diagnosis and treatment options for T2-low asthma has proposed NA as a prototypical T2-low, aging-related asthma phenotype, suggesting a conceptual integration between immune aging and T2-low pathophysiology (11). In our own previous transcriptomic analyses (12), gene sets associated with Huntington's disease, Parkinson's disease, and cellular senescence were selectively enriched in the NA subgroup, but not in eosinophilic (EA) or paucigranulocytic asthma (PGA) phenotypes. Among the differentially expressed genes (DEGs) identified between NA and healthy controls, 32 were associated with aging-related gene sets, whereas only 12 aging-related DEGs were found in the EA group, and none were detected in the PGA group (12). These data collectively suggest that NA may be mechanistically linked to aging and age-associated molecular programs, exhibiting a stronger transcriptional connection to senescence compared to other asthma subtypes.

## Aging-driven vicious cycle of neutrophil chemotaxis

Within the 32 aging-related DEGs identified between NA and healthy controls, we found that the neutrophil-recruiting chemokine axis—CXCL8, CXCL1, and their receptor CXCR2—as well as the key inflammatory mediators CCL20 and NLRP3, were significantly upregulated in the airways of NA patients (12). Previous studies have established that in NA, Th17-derived IL-17 induces bronchial epithelial cells and subepithelial fibroblasts to release CXCL8 and CXCL1, thereby promoting neutrophil infiltration into the airway. Given that this pro-neutrophilic IL-17 signaling pathway is aberrantly activated in aging-associated inflammatory microenvironments (13, 14), we hypothesize that aging-induced hyperactivation of the IL-17 pathway serves as a key mechanism linking cellular senescence to the sustained neutrophilic inflammation observed in NA, ultimately establishing a persistent neutrophil chemotactic axis. Notably, the IL-17A–CXCL1/CXCL8–CXCR2 inflammator*y* axis has been implicated in aberrant neutrophil recruitment in several diseases, including Kawasaki disease, chronic obstructive pulmonary disease (COPD), psoriasis, and various cancers (15–19). These underscore that the IL-17 axis drives a self-perpetuating cycle of neutrophil recruitment, highlighting the need for further investigation.

Building upon this chemotactic cycle, the concurrent upregulation of CCL20 provides a critical link for further inflammatory amplification. As a prominent SASP factor, CCL20 acts as a key chemokine for Th17 cell recruitment, facilitating the accumulation of Th17 cells in the airway. This recruitment enhances local IL-17 production, which in turn further stimulates epithelial cells to secrete CXCL8 and CXCL1, thereby creating a positive feedback loop between Th17 cells and neutrophils—a mechanism that has also been described in other inflammatory diseases (20). Beyond its role in chemotaxis, CCL20 serves as both a facilitator of NLRP3 inflammasome activation and a pivotal response gene within its regulatory network (21). Furthermore, by engaging the CCR6/CCL20 axis, it actively recruits Th17 cells to amplify IL-1β–mediated proinflammatory responses (22).

Taken together, we propose that the aging-associated inflammatory microenvironment may promote the pathological amplification of the Th17–neutrophil axis via aberrant IL-17 signaling, thereby contributing to the development and progression of neutrophilic asthma.

## An aging-linked NLRP3-CCL20 axis fuels glucocorticoid-refractory NA

The glucocorticoid-refractory state represents the primary therapeutic challenge in NA. In the aging microenvironment, the accumulation of damage-associated molecular patterns (DAMPs) persistently activates the NLRP3 inflammasome, driving it to become a central effector of immunosenescence and age-related inflammation (23). This senescence-driven activation of NLRP3 is closely associated with the pathogenesis of NA. Dysregulated activation of the NLRP3/caspase-1 pathway markedly enhances the maturation and release of IL-1β. In turn, IL-1β synergizes with IL-6 and TGF-β to activate the STAT3 signaling axis, promoting Th17 cell expansion. This NLRP3–Th17–neutrophil axis, via an IL-1β-dependent mechanism, disrupts airway epithelial barrier integrity and contributes to airway remodeling and fibrosis (24, 25). Experimental evidence further demonstrates that the NLRP3 activator nigericin significantly exacerbates pulmonary neutrophil infiltration (26). In glucocorticoid-refractory NA models, NLRP3/IL-1β signaling is markedly upregulated (27). In NA patients, neutrophils can release neutrophil extracellular traps (NETs) through NETosis, which trigger inflammatory mediator release and immune cell recruitment, leading to further epithelial injury, airway hyperresponsiveness, and mucus secretion. Consequently, this process accelerates disease progression and reduces the therapeutic efficacy of conventional glucocorticoids (28). Notably, while the NLRP3 inhibitor MCC950 demonstrated significant efficacy in alleviating glucocorticoid-refractory NA, its development was curtailed by safety concerns. Encouragingly, next-generation agents are showing great promise: VTX2735 is currently under Phase 2 clinical evaluation, and the orally active OLT1177 (dapansutrile) has exhibited a favorable safety profile, further underscoring the potential of targeting the NLRP3 inflammasome to overcome glucocorticoid refractoriness in NA (29). In our DEG analysis comparing NA patients and healthy controls, we found that NLRP3, IL6, STAT1, and TNFAIP3 were significantly upregulated in the airways of NA patients. Importantly, the diagnostic performance of NLRP3 was substantial, with an AUC of 0.81, highlighting its potential as a biomarker for neutrophilic inflammation and possibly as a predictor of therapeutic response.

In addition, the persistent upregulation of CCL20 further reinforces a glucocorticoid-refractory state (30). By simultaneously facilitating NLRP3 inflammasome activation and expanding the Th17-mediated inflammatory pool, we posit that CCL20 acts in concert with the NLRP3 axis to exacerbate steroid-refractory pathology. Consequently, the CCL20–CCR6 axis represents an additional therapeutic target, with the CCR6 antagonist IDOR-1117-2520 currently undergoing Phase 1 trials.

Taken together, these findings suggest that the accumulation of senescent cells may drive persistent activation of the NLRP3 inflammasome, promoting IL-1β maturation and release via caspase-1. In concert with IL-6 and TGF-β, this facilitates Th17 polarization. Th17-driven T2-low inflammation underlies glucocorticoid responsiveness and amplifies airway neutrophilic infiltration. Meanwhile, the NLRP3–IL-1β axis promotes NETosis, directly damaging bronchial epithelium and attenuating glucocorticoid efficacy. The SASP factor CCL20 recruits additional Th17 cells through the CCR6/CCL20 axis and exerts intrinsic glucocorticoid resistance, thereby generating a cascade amplification. Ultimately, this forms a cycle of “cellular senescence–NLRP3 activation–IL-1β/IL-6/CCL20 upregulation–Th17 polarization/NETosis–glucocorticoid-refractory inflammation.” This mechanism highlights that targeting the “senescence–NLRP3 inflammasome axis” (e.g., via NLRP3 inhibitors or CCL20 blockade) may provide a promising therapeutic strategy to overcome the current challenges in treating glucocorticoid-refractory NA.

## Limitations and future perspectives

The incomplete understanding of NA pathogenesis—which hampers the identification of definitive therapeutic targets—is largely rooted in inherent limitations within the existing research landscape. First, current murine HDM models, primarily designed for T2-high eosinophilic inflammation, fail to replicate the systemic metabolic and aging-driven features of NA. This inability to model the progression toward a steroid-refractory neutrophilic state contributes to a significant translational gap between preclinical findings and clinical reality (31). Second, the understanding of NA is constrained by its inherent etiological heterogeneity. Beyond the inflammaging-driven pathways highlighted in our analysis, other drivers—including obesity, cigarette smoking, and recurrent infections—significantly contribute to NA; however, they are often studied in isolation, with a notable lack of research elucidating their convergence with aging-associated pathways. This fragmentation obscures the molecular drivers of NA, impeding a comprehensive understanding of the disease. Furthermore, sex-specific differences in airway senescence kinetics remain largely overlooked, as current research cohorts frequently lack the sex-stratified analysis needed to define the influence of biological sex on disease progression. Addressing these limitations will require a shift toward multi-omic integration and integrated physiological modeling—a holistic framework essential for unifying these disparate clinical drivers and advancing precision therapeutic strategies.

Crucially, the clinical viability of targeting cellular senescence in airway disease is gaining momentum. Previous research has extensively characterized epithelial senescence and the resulting SASP as key drivers of chronic lung disease, providing a conceptual basis for extending senotherapy to NA (32, 33). Moreover, multiple trials of the senolytic cocktail dasatinib plus quercetin in patients with idiopathic pulmonary fibrosis (IPF), which have demonstrated clinical safety and functional improvement (34, 35). Despite distinct etiologies, NA and IPF share a common senescence-driven inflammatory microenvironment, thus, translating senolytic strategies to NA represents a promising, mechanism-based approach to dismantling this microenvironment and reversing the chronic inflammatory remodeling characteristic of the NA airway.

## Conclusion

In summary, our transcriptomic analysis highlights senescence as a central driver of NA, converging on aberrant IL-17 signaling, NLRP3 inflammasome activation, and CCL20-driven Th17 cell recruitment. Together, these pathways establish a vicious cycle of neutrophil chemotaxis, activation, and glucocorticoid-refractory inflammation, offering a unifying explanation for the chronic and refractory nature of NA.

Beyond these mechanisms, we also observed a marked upregulation of superoxide dismutase 2 (SOD2), an antioxidant enzyme whose dysregulation is linked to mitochondrial redox imbalance. Intriguingly, prior studies have shown that LPS-stimulated neutrophils release extracellular vesicles enriched in mitochondrial SOD2, potentially accounting for its elevation in NA (36). Supporting this, functional enrichment of NA-aging overlapping genes revealed strong associations with cellular response to lipopolysaccharide and bacterial-origin molecules, whereas such enrichment was absent in EA. This suggests that microbial-derived signals, particularly LPS, act as aging-specific drivers of NA. Notably, the elevated expression of the LPS receptor CD14 further underscores its potential role in sustaining this inflammatory circuit.

Collectively, these findings position the senescence–inflammation axis as a central orchestrator of NA and underscore the need to explore targeted interventions-such as NLRP3 inhibitors, CCL20 blockade, or LPS–CD14 modulation-to break the vicious cycle of neutrophilic inflammation and overcome steroid resistance in this challenging asthma phenotype (Graphical abstract).

## References

[1] Subedi S Guntipally M Suwal N Thapa R Bashyal S Panth N. Cellular senescence in chronic obstructive pulmonary disease: molecular mechanisms and therapeutic interventions. Ageing Res Rev. (2025) 110:102813. 10.1016/j.arr.2025.102813 40571131

[2] International Consortium to Classify Ageing-Related Pathologies (ICCARP) respiratory working group members, Weight CM Tatler AL Huckstepp RTR Adcock IM Barnes PJ. The crucial role of normative ageing in respiratory pathogenesis. Lancet Healthy Longev. (2026) 7(4):100842. 10.1016/j.lanhl.2026.100842 42044655

[3] Perez-Garcia J Khodasevich D De Matteis S Rice MB Needham BL Rehkopf DH. Epigenetic age among US adults with chronic respiratory diseases: results from NHANES 1999–2002. Eur Respir J. (2025) 65(6):2500293. 10.1183/13993003.00293-2025 40441879 PMC12177333

[4] Chakraborty A Yadav S Kumar A. Chronic lung diseases (asthma and COPD) among middle-aged and older populations in India: social, individual, and household determinants and their associations with geriatric syndromes. Arch Public Health. (2025) 83(1):186. 10.1186/s13690-025-01675-4 40660407 PMC12257702

[5] Sun J Fan G Hu L Qu ZH Jiang H. Epigenetic age acceleration and allergic diseases: a bidirectional two-sample Mendelian randomization study. Clin Epigenetics. (2025) 17(1):117. 10.1186/s13148-025-01927-8 40618086 PMC12228153

[6] Soma T Nagata M. Immunosenescence, inflammaging, and lung senescence in asthma in the elderly. Biomolecules. (2022) 12(10):1456. 10.3390/biom12101456 36291665 PMC9599177

[7] Ozcan A Boyman O. Mechanisms regulating neutrophil responses in immunity, allergy, and autoimmunity. Allergy. (2022) 77(12):3567–83. 10.1111/all.15505 36067034 PMC10087481

[8] Busse PJ Birmingham JM Calatroni A Manzi J Goryachokovsky A Fontela G. Effect of aging on sputum inflammation and asthma control. J Allergy Clin Immunol. (2017) 139(6):1808–e1806. 10.1016/j.jaci.2016.09.015 27725186 PMC5385166

[9] Crisford H Sapey E Rogers GB Taylor S Nagakumar P Lokwani R. Neutrophils in asthma: the good, the bad and the bacteria. Thorax. (2021) 76(8):835–44. 10.1136/thoraxjnl-2020-215986 33632765 PMC8311087

[10] Fu T Liu J Wang W Li Y Wang Y Cui L. Similarities and differences in kinetic characteristics of airways inflammation, formation of inducible bronchial-associated lymphoid tissue and remodeling between young and old murine asthma surrogates induced with house dust mite. Exp Gerontol. (2023) 175:112160. 10.1016/j.exger.2023.112160 37019047

[11] Thomas D Hamada Y Gibson P Brightling CE Castro M Heaney LG. Diagnosis and treatment options for T2-low asthma. J Allergy Clin Immunol Pract. (2025) 13(7):1527–39. 10.1016/j.jaip.2025.04.055 40373870

[12] Liu X Li B Liu S Zong J Zheng X. To investigate the function of age-related genes in different subtypes of asthma based on bioinformatics analysis. Heliyon. (2024) 10(15):e34766. 10.1016/j.heliyon.2024.e34766 39144919 PMC11320208

[13] Sola P Mereu E Bonjoch J Casado-Pelaez M Prats N Aguilera M. Targeting lymphoid-derived IL-17 signaling to delay skin aging. Nat Aging. (2023) 3(6):688–704. 10.1038/s43587-023-00431-z 37291218 PMC10275755

[14] Duan R Jiang L Wang T Li Z Yu X Gao Y. Aging-induced immune microenvironment remodeling fosters melanoma in male mice via gammadelta17-neutrophil-CD8 axis. Nat Commun. (2024) 15(1):10860. 10.1038/s41467-024-55164-3 39738047 PMC11685811

[15] Lin IC Suen JL Huang SK Chou MH Kuo HC Lo MH. Involvement of IL-17 A/IL-17 receptor A with neutrophil recruitment and the severity of coronary arteritis in Kawasaki disease. J Clin Immunol. (2024) 44(3):77. 10.1007/s10875-024-01673-1 38451335 PMC10920475

[16] Ni Q Li G Chen Y Bao C Wang T Li Y. LECs regulate neutrophil clearance through IL-17RC/CMTM4/NF-kappaB axis at sites of inflammation or infection. Mucosal Immunol. (2024) 17(4):723–38. 10.1016/j.mucimm.2024.05.003 38754839

[17] Kong Q Wang B Zhong Y Chen W Sun J Liu B. Modified Bushen Yiqi formula mitigates pulmonary inflammation and airway remodeling by inhibiting neutrophils chemotaxis and IL17 signaling pathway in rats with COPD. J Ethnopharmacol. (2024) 321:117497. 10.1016/j.jep.2023.117497 38048893

[18] Zeng F Du S Wu M Dai C Li J Wang J. The oncogenic kinase TOPK upregulates in psoriatic keratinocytes and contributes to psoriasis progression by regulating neutrophils infiltration. Cell Commun Signal. (2024) 22(1):386. 10.1186/s12964-024-01758-9 39090602 PMC11292866

[19] Wu L Awaji M Saxena S Varney ML Sharma B Singh RK. IL-17-CXC chemokine receptor 2 axis facilitates breast cancer progression by up-regulating neutrophil recruitment. Am J Pathol. (2020) 190(1):222–33. 10.1016/j.ajpath.2019.09.016 31654638 PMC6943375

[20] Zhang C Xu S Hu RJ Liu XH Yue SY Li XL. Unraveling CCL20’s role by regulating Th17 cell chemotaxis in experimental autoimmune prostatitis. J Cell Mol Med. (2024) 28(10):e18445. 10.1111/jcmm.18445 38801403 PMC11129727

[21] Park SY Kang MJ Jin N Lee SY Lee YY Jo S. House dust mite-induced akt-ERK1/2-C/EBP beta pathway triggers CCL20-mediated inflammation and epithelial-mesenchymal transition for airway remodeling. FASEB J. (2022) 36(9):e22452. 10.1096/fj.202200150RR 35916017

[22] Li X Ding W Lu Y Zhu H Bao W Liu Y. An anti-complement homogeneous polysaccharide from Houttuynia cordata ameliorates acute pneumonia with H1N1 and MRSA coinfection through rectifying Treg/Th17 imbalance in the gut-lung axis and NLRP3 inflammasome activation. Acta Pharm Sin B. (2025) 15(6):3073–91. 10.1016/j.apsb.2025.04.008 40654358 PMC12254813

[23] Lee G Lee GS. Potential role of inflammasomes in aging. Int J Mol Sci. (2025) 26(14):6768. 10.3390/ijms26146768 40725015 PMC12296179

[24] Tan HT Hagner S Ruchti F Radzikowska U Tan G Altunbulakli C. Tight junction, mucin, and inflammasome-related molecules are differentially expressed in eosinophilic, mixed, and neutrophilic experimental asthma in mice. Allergy. (2019) 74(2):294–307. 10.1111/all.13619 30267575

[25] Williams EJ Negewo NA Baines KJ. Role of the NLRP3 inflammasome in asthma: relationship with neutrophilic inflammation, obesity, and therapeutic options. J Allergy Clin Immunol. (2021) 147(6):2060–2. 10.1016/j.jaci.2021.04.022 33932467

[26] Zhou L Zhang H Liu L Zhang F Wang L Zheng P. Intermittent hypoxia aggravates asthma inflammation via NLRP3/IL-1beta-dependent pyroptosis mediated by HIF-1alpha signalling pathway. Chin Med J (Engl). (2025) 138(14):1714–29. 10.1097/CM9.0000000000003608 40587235 PMC12273680

[27] Kim RY Pinkerton JW Essilfie AT Robertson AAB Baines KJ Brown AC. Role for NLRP3 inflammasome-mediated, IL-1beta-dependent responses in severe, steroid-resistant asthma. Am J Respir Crit Care Med. (2017) 196(3):283–97. 10.1164/rccm.201609-1830OC 28252317

[28] Tsai CH Lai AC Lin YC Chi PY Chen YC Yang YH. Neutrophil extracellular trap production and CCL4L2 expression influence corticosteroid response in asthma. Sci Transl Med. (2023) 15(699):eadf3843. 10.1126/scitranslmed.adf3843 37285400

[29] Horvat JC Kim RY Weaver N Augood C Brown AC Donovan C. Characterization and inhibition of inflammasome responses in severe and non-severe asthma. Respir Res. (2023) 24(1):303. 10.1186/s12931-023-02603-2 38044426 PMC10694870

[30] Wang L Yang M Wang X Cheng B Ju Q Eichenfield DZ. Glucocorticoids promote CCL20 expression in keratinocytes. Br J Dermatol. (2021) 185(6):1200–8. 10.1111/bjd.20594 34157145 PMC9290737

[31] Matera MG Calzetta L Rinaldi B de Novellis V Page CP Barnes PJ. Animal models of chronic obstructive pulmonary disease and their role in drug discovery and development: a critical review. Expert Opin Drug Discov. (2025) 24:1–20. 10.1080/17460441.2025.2466704 39939153

[32] Barnes PJ. Senotherapy for chronic lung disease. Pharmacol Rev. (2025) 77(4):100069. 10.1016/j.pharmr.2025.100069 40554265 PMC12405932

[33] Devulder JV Fenwick PS Kolosionek E Al-Sahaf M Viola P Lemaire R. Clearance of senescent cells by BCL(XL)-PROTAC: a novel approach to treat COPD? Aging Cell. (2026) 25(4):e70487. 10.1111/acel.70487 41979382 PMC13078126

[34] Nambiar A Kellogg D III Justice J Goros M Gelfond J Pascual R. Senolytics dasatinib and quercetin in idiopathic pulmonary fibrosis: results of a phase I, single-blind, single-center, randomized, placebo-controlled pilot trial on feasibility and tolerability. EBioMedicine. (2023) 90:104481. 10.1016/j.ebiom.2023.104481 36857968 PMC10006434

[35] Zhu Y Prata L Gerdes EOW Netto JME Pirtskhalava T Giorgadze N. Orally-active, clinically-translatable senolytics restore alpha-Klotho in mice and humans. EBioMedicine. (2022) 77:103912. 10.1016/j.ebiom.2022.103912 35292270 PMC9034457

[36] Bao W Xing H Cao S Long X Liu H Ma J. Neutrophils restrain sepsis associated coagulopathy via extracellular vesicles carrying superoxide dismutase 2 in a murine model of lipopolysaccharide induced sepsis. Nat Commun. (2022) 13(1):4583. 10.1038/s41467-022-32325-w 35933512 PMC9357088

